# Response to Kruse-Plass et al. (2017) regarding the risk to non-target lepidopteran larvae exposed to pollen from one or more of three Bt maize events (MON810, Bt11 and 1507)

**DOI:** 10.1186/s12302-017-0119-8

**Published:** 2017-05-10

**Authors:** Joe N. Perry, Paolo Barberi, Detlef Bartsch, A. N. E. Birch, Achim Gathmann, Jozsef Kiss, Barbara Manachini, Marco Nuti, Stefan Rauschen, Joachim Schiemann, Mechthild Schuppener, Jeremy Sweet, Christoph C. Tebbe, Fabio Veronesi

**Affiliations:** 1 Oaklands Barn, Lug’s Lane, Broome, Norfolk, NR35 2HT UK; 2 Institute of Life Sciences, Scuola Superiore Sant’Anna, Pisa, Italy; 3 Department Genetic Engineering, BVL-Bundesamt für Verbraucherschutz und Lebensmittelsicherheit, Berlin, Germany; 4 Ecological Sciences, The James Hutton Institute, Dundee, Scotland UK; 5 Department of Plant Protection Products, BVL-Bundesamt für Verbraucherschutz und Lebensmittelsicherheit, Brunswick, Germany; 6 Plant Protection Institute, Szent István University, Gödöllő, Hungary; 7 Scienze e Tecnologie Biologiche Chimiche e Farmaceutiche, Università degli Studi di Palermo, Palermo, Italy; 8 Scuola Superiore Sant’Anna, Pisa, Italy; 9 Forschungszentrum Jülich GmbH, Projektträger Jülich, Jülich, Germany; 10 Institute for Biosafety in Plant Biotechnology, Julius Kühn-Institut, Federal Research Centre for Cultivated Plants, Quedlinburg, Germany; 11 i-bio Information Biowissenschaften, Aachen, Germany; 12 JT Environmental Consultants Ltd, 6 The Green, Willingham, Cambridge, CB24 5JA UK; 13 Thünen Institute of Biodiversity, Federal Research Institute for Rural Areas, Forestry and Fisheries, Brunswick, Germany; 14 Dipartimento di Scienze Agrarie, Alimentari ed Ambientali, Università degli Studi di Perugia, Perugia, Italy

**Keywords:** Genetically modified organisms, Environmental risk assessment, Exposure, Host plants, Non-target organisms, Lepidoptera, Pollen deposition

## Abstract

We respond to the paper of Kruse-Plass et al. (Environ Sci Eur 29:12, 2017), published in this journal, regarding the risk to non-target lepidopteran larvae exposed to pollen from one or more of three Bt maize events (MON810, Bt11 and 1507). We emphasise that what is important for environmental risk assessment is not the number of pollen grains per se, but the degree of exposure of a NT lepidopteran larva to Bt protein contained in maize pollen. The main text of this response deals with general issues which Kruse-Plass et al. have failed to understand; more detailed refutations of each of their claims are given in Additional file 1. Valid environmental risk assessment requires direct measurement of pollen on leaves at varying distances outside a source field(s); such measurements reflect the potential exposure experienced by an individual larva on a host plant. There are no new data in the Kruse-Plass et al. paper, or indeed any data directly quantifying pollen on actual host-plant leaves outside a maize field; only data gathered within or at the edge of maize crops were reported. Values quoted by Kruse-Plass et al. for deposition on host plants outside the field were estimates only. We reiterate the severe methodological criticisms made by EFSA [Relevance of a new scientific publication (Hofmann et al. 2016) for previous environmental risk assessment conclusions and risk management recommendations on the cultivation of Bt-maize events MON810, Bt11 and 1507. EFSA Supp Publ; EN-1070, 2016], which render this estimation procedure unreliable. Furthermore, criticisms of EFSA (EFSA J 2015(13):4127, 2015) and of EFSA [Relevance of a new scientific publication (Hofmann et al. 2016) for previous environmental risk assessment conclusions and risk management recommendations on the cultivation of Bt-maize events MON810, Bt11 and 1507. EFSA Supp Publ; EN-1070, 2016] made by Kruse-Plass et al. are shown in Additional file 1 to be without foundation. We therefore consider that there is no valid evidence presented by Kruse-Plass et al. to justify their conclusions.

**Electronic supplementary material:**

The online version of this article (doi:10.1186/s12302-017-0119-8) contains supplementary material, which is available to authorised users.

## Background

We respond to Kruse-Plass et al. [1] who commented on EFSA [2, 3] and on several of our previous publications (references to Perry et al. in [2, 3]), regarding the risk to non-target (NT) lepidopteran larvae of pollen from one or more of three Bt maize events (MON810, Bt11 and 1507). The trait in the Bt maize is insect resistance designed to act against particular target lepidopteran pests through Bt protein expressed throughout the maize plant. The risk of an adverse environmental effect occurs through Bt maize pollen deposition on the leaves of the host plants of those susceptible NT lepidopteran larvae and subsequent unintended ingestion of pollen by larvae at a sensitive life stage. The EFSA publications [2, 3] were particularly concerned to quantify the risk to Lepidoptera of conservation concern in protected habitats. The main text of this response deals with general issues which Kruse-Plass et al. have failed to understand; more detailed refutations of each of their claims are given in Additional file 1.

## Main text

Kruse-Plass et al. focus largely on the amount of pollen deposition as measured in mechanical samplers using data presented by Hofmann et al. [4, 5], but what is important for environmental risk assessment (ERA) is not the number of pollen grains per se, but the degree of exposure of a NT lepidopteran larva to Bt protein contained in maize pollen. All such risk assessments are completed by considering the toxicity of the Bt protein, contained within the pollen, towards the exposed life stage of a given NT species.

Toxicity and the difference between actual and potential toxicity follow a chronological process: firstly maize pollen shed, then maize pollen movement through the air, deposition onto a host-plant leaf, possible loss through removal from that leaf, redistribution on that leaf, degradation of the Bt protein within the pollen grain, ingestion by a NT lepidopteran larva, and finally a potential toxic effect on that larva. In practice, the factors driving this process act to limit the actual exposure to toxic maize pollen.

This is the basis for the factors described in Appendix A and Table 2 of the EFSA Opinion [3]:

(i) Pollen grain density as measured in mechanical samplers in and outside a maize field differs from, and is usually considerably greater than, pollen density as measured on actual host plants because: (a) host plants are three-dimensional structures with randomly oriented leaves (not fully horizontal) on which pollen may not adhere, and (b) wind and rain act to further remove that pollen which does adhere from leaves.
(ii) Lack of synchrony between the period of Bt maize pollen deposition and the life stage of the NT species concerned will reduce the amount of pollen ingested. In some cases, if the phenology of the NT larva does not coincide with the period of maize flowering and pollen shed then there will be no ingestion of pollen.
(iii) Pollen ingestion on a leaf by a lepidopteran larva may be reduced through pollen consumption by other non-lepidopteran species and/or by the displacement of pollen into aggregated areas within a leaf; avoidance of such high-density areas has been observed for such larvae.
(iv) Toxicity following ingestion by a larva occurs if, and only if, the maize pollen grain is from a lepidopteran-resistant Bt maize plant. Pollen from conventional, non-Bt maize, or from coleopteran-resistant Bt maize is harmless to lepidopteran larvae.
(v) Degradation of the Bt protein within the pollen grain (for grains that have spent an appreciable time between release from a maize plant, subsequent transport to the host plant, and finally ingestion by a larva on that host plant) may reduce the toxicity of the pollen up to the point when the Bt protein is finally released from the ingested maize pollen and finds its way to the specific Bt protein receptor-binding sites in the larvae midgut epithelium.

It can be seen that at each stage of this process, the apparent density and potential toxicity of pollen grains to NT Lepidoptera as measured on leaves may be reduced compared with mechanical samplers.

Furthermore, because exposure as experienced by an individual NT larva is what is important in risk assessment, there is no merit in the argument of Kruse-Plass et al. that “mean leaf measurements of single plants or days are not representative” and that “leaf pollen deposition over the flowering period must be considered”. On the contrary, measurements of pollen on leaves reflect potential exposure directly, transparently and unambiguously. This has been realised for many years by authors such as Darvas et al. [6], Gathmann et al. [7], Schuppener et al. [8] and Masetti et al. [9]. More recently, the meticulous approach of Lang et al. [10] has added extremely valuable data to the literature. Verification of risk assessment requires such direct measurements outside of source fields, not an over-reliance on uncertain standardisation as proposed by Kruse-Plass et al. Whilst the EFSA ERA [2, 3] was based on all the available literature at the time, the Kruse-Plass et al. approach is to use limited data, interpreted selectively.

## Conclusions

We emphasise that there are no data in [4, 5] or in Kruse-Plass et al. [1] that directly quantifies pollen on actual host-plant leaves outside a maize field; only data gathered within or at the edge of maize crops were reported. Values quoted by Kruse-Plass et al. for deposition on host plants outside the field were estimates only. Crucially, we reiterate the severe methodological criticisms made by EFSA in [2], which render this estimation procedure unreliable (see Additional file 1). Furthermore, criticisms of EFSA ERAs [2, 3] made by Kruse-Plass et al. are shown in the Additional file 1 to be without foundation. In summary, there are no new data in the Kruse-Plass et al. publication and none of the accusations they made concerning EFSA [2, 3] have any merit; we therefore disagree with their conclusions.

## Additional file

**Additional file 1**. Additional information.

## Abbreviations

EFSA: European Food Safety Authority
ERA: environmental risk assessment
NT: non-target
