# Supplementary material for: Response to Kruse-Plass et al. (2017) regarding the risk to non-target lepidopteran larvae exposed to pollen from one or more of three Bt maize events (MON810, Bt11 and 1507)
Source: Environ Sci Eur. 2017 May 10;29(1):21. doi: 10.1186/s12302-017-0119-8 (PMC5423918; doi:10.1186/s12302-017-0119-8)
Supplement: Supplementary file 1 — Additional file 1. Additional information. [file 12302_2017_119_MOESM1_ESM.docx]

**Additional File**

In this document we address each section of Kruse-Plass et al. in turn. For convenience, quotations of text from their paper and those of Hofmann et al. are in red; quotations of text from EFSA publications and those of Perry et al. are in purple.

**Kruse-Plass et al.** **section: Background**

The justification mentioned in the Kruse-Plass et al. text “The justification for the original shorter period in the EFSA model was that >92% of pollen shed would take place within the first 7 days.” is not well described. In addition, Perry et al. (2013) stated that a week exceeded the duration of the life-stage for the species of Lepidoptera most commonly studied and modelled (1^st^ instar neonates of *Inachis io*): “Note that the development time of the first and most sensitive instar of the butterfly *Inachis io*, about four days, is considerably less than this.”

The description of the 1 km square protected habitat given in the Kruse-Plass et al. text: “the EFSA Panel introduced a hypothetical 1 × 1 km^2^ nature reserve area to their revised model. Exposure and mortality effects were averaged within this model nature reserve by calculating weighted averages over three distances (for an adjacent nature reserve: 5, 500, 1000 m). Thus, the estimates of exposure and effects were further diminished.” is wrong. The EFSA (2015) Opinion (doi: 10.2903/j.efsa.2015.4127) stated: “...Effectively, this resulted in computing a weighted average mortality for larvae at the following distances from the nearest Bt-maize field: S, S, S, S + 500, S + 500 and S + 1 000.”

Kruse-Plass et al. have seemingly misread the variable *S* as the number 5 and appear to have misunderstood the methodology as a result. The EFSA (2015) Opinion gave expected larval mortality for a wide range of values of *S*, the separation distance to protected habitats, ranging from *S*=5 to *S*=3000. Far from *diminishing* the estimates of exposure, the EFSA (2015) Opinion emphasized the *conservative* nature of the methodology: “this results in a conservative approach (i.e. the assumptions lead to a higher estimated exposure to maize pollen) in which mortality is not underestimated.” For transparency, we explain briefly as follows. The six values S, S, S, S + 500, S + 500 and S + 1 000, derive from the aggregation of two scenarios: (i) the usual case where three larvae at the nearest edge, centre and farthest edge of the protected habitat from the nearest maize field are all more than S + 1000 from the next nearest maize field, yielding values S, S + 500 and S + 1000; (ii) a worst-case where two maize fields are the same distance, S, from the protected habitat but on opposite sides of it, where the three larvae yield values of S, S+500 and S.

The description of the conclusion reached by the EFSA GMO Panel in the 2015 Opinion given by Kruse-Plass et al. is wrong. They claimed the conclusion was: “that no changes to the previously proposed 20–30 m buffer between Bt-maize cultivation and protected areas would be required under ‘realistic’ or even ‘worst case’ assumptions”. This was the case for the maize MON810 and Bt11 events: “Under the most realistic and even conservative scenarios, the estimated mortality for all species considered is always less than 0.5 % for the previously recommended isolation distance of 20 m. Therefore, the previous EFSA GMO Panel recommendation for isolation distances around protected habitats, within which maize MON 810/Bt11 should not be cultivated, remains valid.”, but not for the maize 1507 event, for which the conclusion was: “the previously recommended isolation distance of 30 m from the nearest field of maize 1507 would still protect NT Lepidoptera with known levels of sensitivity, including the ‘highly-sensitive’ *Plutella xylostella*. Should hypothetical species with greater sensitivities exist, larger isolation distances would be needed to ensure the desired level of protection. The EFSA GMO Panel provides risk managers with a tool to estimate and mitigate the risk for NT Lepidoptera of conservation concern, considering the above-mentioned three scenarios [i.e. direct comparison, most realistic and conservative] at a range of isolation distances, at two protection levels and for lepidopteran species with a wide spectrum of sensitivities to Bt toxins, including hypothetical species not yet assessed. This will allow risk managers to select the most appropriate risk management measures (i.e. isolation distances) that are proportionate to the level of risk identified according to appropriate protection goals.” Hence, the conclusion reached by EFSA (2015) was more nuanced than the impression given by Kruse-Plass et al. Furthermore, EFSA (2015) recommended further research into the sensitivity of NT Lepidoptera to Bt-maize pollen and to determine whether or not “NT lepidopteran larvae, with an ‘extremely high’ sensitivity to the Cry1Ab protein, are present and feeding on host plants occurring in and adjacent to maize fields at the time of pollen shed.“

Any reference to Holst et al. (2013) (reference 18 of Kruse-Plass et al.) should be accompanied by the information in the response by Perry et al. (2013) (their reference 17, incorrectly spelt), which exposed the paucity of their arguments, and which also drew together much useful data on toxicity and deposition in a review of available literature.

**Kruse-Plass et al.** **section: Comparing the EFSA scenarios for the dose–distance relationship of *Urtica* leaf pollen density with the empirical findings of Hofmann et al.**

The y-axis label of Figure 1 of Kruse-Plass et al. gives the false impression that Hofmann et al. (2016) measured pollen on actual *Urtica* leaves at distances from 0.5m to 4.4km outside a maize field. By contrast, as EFSA (2016) (<https://www.efsa.europa.eu/en/supporting/pub/en-1070>) made clear, Hofmann et al. (2016) measured actual pollen density on the leaves of plants of various species, including nettles, only *within maize crops* and *at field edges*. Unlike Lang et al. (2015), no data were presented that would enable verification of pollen deposition assumptions on host plants in protected habitats, outside maize fields. Instead, the blue data points in Figure 1 of Kruse-Plass et al. are all estimates, i.e. processed data and not measured values, of pollen on host-plant leaves. Furthermore, the estimation procedure used is biased (see below).

Figure 1 of Kruse-Plass et al. and associated text compares results of Hofmann et al. (2016) with those of EFSA (2015) on the basis of different units: the former is based on what Hofmann et al. (2014) described as the “whole maize flowering period ... (3 to 5 weeks and sometimes longer)”, the latter is based on one week. Direct comparisons are therefore misleading (and see below).

Kruse-Plass et al. claim: “We further note that in Fig. 1 in EFSA (2016), the dose–distance relationship ‘EF’ does not relate to the ‘most realistic’ scenario of the EFSA Panel, although it is implied to do so. The factor applied (0.396) is different from that of the original MR scenario in EFSA (2016) (0.0376). The EF line for the EFSA Panel MR scenario should correctly be 10 times lower and would thus lie below the leaf density data of Lang et al. (2015) as shown in Fig. 2.”

Kruse-Plass et al. have misunderstood EFSA (2016), although it is difficult to understand how they could have done so, since the reasoning is spelt out transparently in section 3.2.1:

“Lang et al. (2015) measured actual pollen density on nettle leaves at various distances from a source maize field. Their data enabled the GMO Panel to test some of the assumptions regarding the second and third exposure factors in EFSA (2015), namely the effect of 3-D structure of leaves and the effect of wind and rain removing pollen. The estimated values for these factors (see Table 2 and Appendix A of EFSA, 2015) were, respectively, 0.61 and 0.65, so their product is 0.396. Under the EFSA (2015) DC scenario, the Lang et al. (2015) data would be expected to follow the Hofmann et al. (2014) dose-distance relationship. Under the EFSA (2015) MR scenario, the Lang et al. (2015) data would be expected to follow the Hofmann et al. (2014) dose-distance relationship, reduced by this multiplicative factor of 0.396.”

EFSA (2016) correctly identified that the Lang et al. (2015) data “enabled the GMO Panel to test some of the assumptions regarding the second and third exposure factors in EFSA (2015), namely the effect of 3-D structure of leaves and the effect of wind and rain removing pollen”. These factors, described above in our General Comments under (i), are the ones that could be included in a fair comparison of the EFSA and Hofmann approaches. It is to be expected that the actual exposure through ingestion of toxic pollen by a NT lepidopteran larva would indeed be smaller than that implied by the line EF in Figure 1 of EFSA (2016), but there is no information to determine by how much. The comparison between the lines AB, CD and EF is therefore fair and transparent. There is therefore no validity in the implications by Kruse-Plass et al., at the end of this section and in their Figure 2, concerning the most-realistic scenario and the line EF. Furthermore, the blue line in their Figure 2, labelled ‘best fit Lang et al. 2015’, is irrelevant, as the hyperbolic function shown (y = 0.95 + 25.54/x) is not a good descriptor of pollen deposition: it implies infinite deposition at the edge of fields where x=0 and deposition that has a minimum value of 0.95 no matter how far from the edge of the field deposition is measured. Hyperbolic functions such as this all suffer from the drawback (and see EFSA, 2015 in relation to a similar problem with extrapolations from the Hoffman et al. formulation) that they imply that the source field(s) emit an infinite amount of pollen.

**Kruse-Plass et al.** **section: Relationship of standardised pollen deposition to leaf pollen density**

There is nothing in this section beyond a restatement of the Hofmann (2014) and (2016) position.

**Kruse-Plass et al.** **section: Consistency of the dose–distance relationship**

The only new information in this section is an explanation by Kruse-Plass et al. that under the Hofmann et al. approach, the value 0.2m is taken to represent all in-field distances from the nearest source of pollen. The steps in the Kruse-Plass et al. argument by which their Figure 1 is constructed therefore appear to be: (i) assume (on the basis of no presented evidence) that the Hofmann et al. (2014) regression derived for mechanical PMF pollen samplers applies inside field to the values of pollen sampled on leaves (grey points in their Figure 1); (ii) use that regression to standardize all data sampled from leaves at varying distances within the field to values expected if data had been sampled from leaves within the field at 0.2m from the field edge (although the grey points appear to show no trend with distance from the edge); (iii) compare these values from leaf deposition with those from mechanical PMF pollen samplers also collected within the field at 0.2m from the field edge, giving a multiplier of 0.68 for nettle plants; (iv) apply this multiplier to all of the mechanically sampled PMF values taken outside the field to standardize them to values expected if they were leaf deposition data; (v) assume that the distance from any plant within the field to the nearest source of pollen can be represented by an arbitrary distance of 0.2m; (vi) amend the corresponding x-axes labels in the Hofmann (2014, 2016) publications from “within field”’ or “In field close to”, respectively, to read “Distance to next pollen source in m”; and finally (vii) present the y-axis label of Figure 1 as if all the data were actual pollen deposition on leaves, although the great majority do not come from leaves but are the standardized estimates from steps 1-8. Such a byzantine approach to estimation through standardization may be appropriate in disciplines such as physics where variability may be limited. In our experience, based on extensive field work, biological parameters are best measured directly, which is why we insist that pollen density of host-plant leaves far from the maize pollen source should be measured unambiguously, on leaves of that host-plant.

Perry et al. (2013) extracted data from 14 studies from the literature, representing over 50 datasets. Pollen deposition from 42 replicate in-field measurements and 24 replicate measurements at the edge of maize fields gave a ratio of 3.09 : 1 for pollen deposition in-field : edge, a value that corresponded well with several well-known studies on the NT Monarch butterfly (e.g. Pleasants et al., 2001) that reported that pollen deposition within a maize crop is approximately 2.7 times that at the edge.

The exact relationship between within-field pollen deposition, deposition at the edge of the field and deposition outside of the field is currently unknown. However, it cannot be assumed that all positions at the edge of a field and within it can be reduced to a single value (0.2m, or any other value). Indeed, if that *were* the case then no difference would be expected between the edge and the inner part of the field; since the 3.09 in-field : edge ratio from the literature clearly implies a substantial difference exists, the Hofmann et al. approach is deficient.

In addition, we note that the situation within a maize canopy within a field, where there are relatively very few host-plants for NT lepidopteran larvae and those that do exist within a field are usually close to the edge and may be heavily shaded by the maize canopy, is very different from that outside the field, where host-plants are more abundant and usually not as shaded by other plants.

**Kruse-Plass et al.** **section: Supporting information**

There is nothing new in this section; it represents a restatement of the Hofmann et al. (2014) and (2016) position.

**Kruse-Plass et al.** **section: Sampling method**

EFSA (2016) noted that the method used by Hofmann et al. (2014; 2016) for estimating the density of pollen on individual leaves does not employ random sampling, but is designed to deliberately include areas of high pollen density on leaves, resulting in statistically biased overestimates of pollen deposition. Statistical bias occurs when the expected value of an estimate differs from the true underlying value of the parameter being estimated. Random sampling produces statistically unbiased estimates, although as Kruse-Plass et al. note, it is not the only way of obtaining unbiased samples. Kruse-Plass et al. claim that the Hofmann et al. sampling method, by which pollen deposition densities per leaf were measured, was not biased, citing as evidence a reiteration of published material. However, that very material (Table 1 of Hofmann et al., 2011) states that their chosen method gave estimates that were on average over two times greater (130:64) than the unbiased estimates obtained through random sampling.

**Kruse-Plass et al.** **section: Standardised methods and data**

Hofmann et al. (2016) stated that “using the regression function for pollen deposition all measurements were adjusted so that their values corresponded to a within-field location near the pollen source (0.2 m). For instance, measurements taken at a 1-m distance were adjusted by a conversion factor of 2.57.” EFSA (2016) noted that this standardization involved “potential multiplication or division by five-fold or more, using a relationship with no evidential basis for the data on which it is used”. We repeat that this standardisation is unnecessary; information should be given which facilitates identification of the relationship between pollen deposition dose and distance. Within-field deposition cannot just be assumed to obey the same dose-distance relationship as that which holds outside the field without additional evidence (and see above).

**Kruse-Plass et al.** **section: Exposure of protected habitats**

EFSA Opinions have never sought to imply that there is *no* exposure in protected habitats. Instead, environmental risk assessment methodology attempts to estimate the exposure and subsequent mortality of NT Lepidoptera within such habitats.

**Kruse-Plass et al.** **section: No need for model revision?**

The first paragraph of this section contains a lengthy quote from EFSA (2016). The next two paragraphs represent a highly obfuscatory piece of sophistry by Kruse-Plass et al., based on several unfair comparisons. Specifically, the proper comparisons were given in Section 3.2.2 of EFSA (2015): “At the edge of the field exactly, the estimated dose under the Perry et al. (2010, 2011, 2012, 2013) dose–distance relationship [i.e. the dose used in EFSA Opinions up until that time] is 221.8 pollen grains/cm^2^ per week. The dose used for the Hofmann et al. (2014) dose–distance relationship ... can be seen, from Figure 3 of Hofmann et al. (2014), to be between 48 and 89 pollen grains/cm^2^ per week. Within the field, the dose used for the Perry et al. (2010, 2011, 2012, 2013) dose-distance relationship is 2.76-fold higher than the dose used at the edge, i.e. 611.6 pollen grains/cm^2^ per week. The estimated dose under the Hofmann et al. (2014) dose–distance relationship, from their Figure 3, is about 89 pollen grains/cm^2^ per week”. The fourth paragraph takes no account of the fact that EFSA (2015) refined its model predictions, accounting for newly reported information in Hofmann et al. (2014). Under its Direct Comparison scenario EFSA (2015) used an estimated dose of 5.5 pollen grains / cm^2^ per week for distances 20m from maize fields and an estimated dose of 4.3 pollen grains / cm^2^ per week for distances 30m from maize fields, instead of the values of 0.28 and 0.01, respectively, as claimed by Kruse-Plass et al.

There is no valid evidence presented by Kruse-Plass et al. to warrant their claims made in this section.

**Kruse-Plass et al.** **section: Variability of leaf deposition data**

Regarding between-site variability, EFSA (2015) states: “The third, ‘conservative’ scenario ... accounts for between-sample variability. ... The EFSA GMO Panel emphasises that caution is required in the interpretation of this scenario, because for every site-occasion for which exposure is nine-fold higher than the expected value, there will be a site-occasion for which exposure is nine-fold lower than expected”. Regarding within-site variability, little is known of the effects of aggregation of pollen into clumped between-plant distributions, although there is evidence that NT lepidopteran larvae avoid within-leaf aggregations (for references, see Appendix A of EFSA, 2015). Ignoring that avoidance effect, Perry et al. (2010) studied the effects of aggregated pollen distribution, such that exposure was much greater on some host-plant leaves than on others, whilst maintaining the same overall amount of pollen. Perry et al. (2010) stated: “stochastic simulations suggested that the increase in mortality rates owing to aggregation of pollen deposition into heterogeneous clumps will be no more than one-third of those [deterministic simulations] reported.” Hence despite the non-linear nature of the mortality-dose relationship, outcomes for expected mortality are not expected to differ greatly. Both EFSA (2015) and EFSA (2016) acknowledged variability documented in this section by Kruse-Plass et al. that could give larger-than-average values for exposure. However, EFSA (2016) states, transparently: “the GMO Panel has always taken a deterministic approach, basing their recommendations on estimates of mortality averaged over populations of NT Lepidoptera with appropriate exposure.”

**Kruse-Plass et al.** **section: Conclusions**

There is nothing new in this section; it represents a restatement of the Hofmann et al. (2014) and (2016) position.

There is one sentence with which we agree, which is where Kruse-Plass et al. state: “In our opinion, to fulfil the requirements of an environmental risk assessment for Bt-maize cultivation aiming to protect sensitive species in nature reserve areas, deposition levels should be set in accordance with observed data.” However, we disagree with Kruse-Plass et al. on the best way to achieve that.

Since no new data was presented in the Kruse-Plass et al. publication and since none of the accusations they made concerning EFSA (2015, 2016) have any merit, we disagree with their conclusions.

*References in this Additional File not given in main text*

European Food Safety Authority (2011) Scientific opinion updating the evaluation of the environmental risk assessment and risk management recommendations on insect resistant genetically modified maize 1507 for cultivation. EFSA J 9:2429. doi:10.2903/j.efsa.2011.2429

Hofmann F, Otto M, Kuhn U, Ober S, Schlechtriemen U, Vögel R (2011) A new method for in situ measurement of Bt-maize pollen deposition on host-plant leaves. Insects 2:12–21. doi:10.3390/insects2010012

Perry JN, Devos Y, Arpaia S, Bartsch D, Gathmann A, Hails RS, Kiss J, Lheureux K, Manachini B, Mestdagh S, Neemann G, Ortego F, Schiemann J and Sweet JB, 2010. A mathematical model of exposure of non-target Lepidoptera to Bt-maize pollen expressing Cry1Ab within Europe. Proceedings of the Royal Society B: Biological Sciences, 277, 1417–1425.

Perry JN, Devos Y, Arpaia S, Bartsch D, Gathmann A, Hails RS, Kiss J, Lheureux K, Manachini B, Mestdagh S, Neemann G, Ortego F, Schiemann J and Sweet JB, 2011. The usefulness of a mathematical model of exposure for environmental risk assessment. Proceedings of the Royal Society B: Biological Sciences, 278, 982–984.

Perry JN, Devos Y, Arpaia S, Bartsch D, Ehlert C, Gathmann A, Hails RS, Hendriksen NB, Kiss J, Messean A, Mestdagh S, Neemann G, Nuti M, Sweet JB and Tebbe CC, 2012. Estimating the effects of Cry1F Bt-maize pollen on non-target Lepidoptera using a mathematical model of exposure. Journal of Applied Ecology, 49, 29–37.

Perry, J.N., Arpaia, S., Bartsch, B., Birch, A.N.E., Devos, Y., Gathmann, A., Gennaro, A., Kiss, J., Messéan, A., Mestdagh, S., Nuti, M., Sweet, J.B. & Tebbe, C.C. (2013) No evidence requiring change in the risk assessment of Inachis io larvae. Ecological Modelling, 268, 103–122. <http://doi.org/10.1016/j.ecolmodel.2013.08.004>

John M. Pleasants, Richard L. Hellmich, Galen P. Dively, Mark K. Sears, Diane E. Stanley-Horn, Heather R. Mattila, John E. Foster, Peter Clark and Gretchen D. Jones (2001) Corn pollen deposition on milkweeds in and near cornfields. Proc. Natl. Acad. Sci., 98, 11919-11924. doi: 10.1073/pnas.211287498
